# Eating Behavior and the Evolutionary Perspective on Anorexia Nervosa

**DOI:** 10.3389/fnins.2019.00596

**Published:** 2019-06-13

**Authors:** P. Södersten, U. Brodin, M. Zandian, C. Bergh

**Affiliations:** Karolinska Institutet, Mandometer Clinic, Huddinge, Sweden

**Keywords:** evolution, anorexia, eating, hypothalamus, brainstem, prefrontal cortex, treatment, mathematical models

## Abstract

On the standard perspective, anorexia nervosa and other eating disorders are caused by genetically determined, neurochemically mediated mental illnesses. Standard treatment, cognitive behavioral therapy (CBT), targets cognitive processes thought to maintain the disorders. Effective neurochemically based treatments are not available and the rate of remission is ≤25% 1 year after CBT, with unknown outcomes in the long-term. With starvation as the major threat in biological history, the evolutionary perspective focuses on foraging for food and eating behavior. A neural network, including hypothalamic arcuate peptide-neurons, brainstem serotonin- and dopamine-neurons and their prefrontal cortical projections, mediates (rather than controls) the behavioral adaptations to variations in food availability; activation of the network is associated with opposing behavioral outcomes depending upon external variations. In the clinic, the control of eating behavior is therefore outsourced to a machine that provides feedback on how to eat. Hundreds of eating disorders patients have recovered by practicing eating; the rate of remission is 75% in on average 1 year of treatment, the rate of relapse is 10% over 5 years of follow-up and no patient has died. A two-parameter asymptotic exponential growth curve modeled the eating behavior of 17 healthy women but not that of 17 women with anorexia nervosa. When in remission, the eating behavior of the anorexic women approached that of the healthy women. It is suggested that the treatment of eating disorders should focus on eating behavior.

## Introduction

“Anorexia nervosa is a psychiatric disorder characterized by fear of weight gain and dangerously low body weight … mortality rate exceeds that of other psychiatric disorders … finding comprehensive brain-based models … has been difficult" (Frank et al., 2018). Thus start most accounts. But it was recently suggested that this standard perspective needs to be modified because the treatment of anorexia is at a standstill (Gutierrez and Birmingham, 2018). We will describe the standard perspective and its translation into clinical practice first and then we will describe the evolutionary perspective, with eating behavior in clinical practice.

## The Standard Perspective

On the standard perspective, anorexia is caused by a pre-existing, neurochemically mediated, genetically determined mental disorder as outlined some time ago as: “We hypothesize that people with anorexia nervosa have a trait-related *increase* in 5-HT neuronal transmission that occurs in the premorbid state and persists after recovery” (Kaye et al., 2003) and: “Childhood anxiety represents one important genetically mediated pathway toward the development of anorexia nervosa and bulimia nervosa” (Kaye et al., 2004). The perspective is similar today (Treasure et al., 2015).

### Clinical Translation of Neurochemistry and Genetics

If anorexia is caused by an increase of 5-HT synthesis an inhibitor or an antagonist should be used, but paradoxically, indirect agonists are used, and although useful in patients with mental disorders (Locher et al., 2017), these drugs are not useful in patients with anorexia (Walsh et al., 2006; Zandian et al., 2007). But neither are other drugs, including neuroleptics, which are valuable for patients with mental disorders (Lieberman et al., 2005), useful in patients with eating disorders (Attia et al., 2019). This differential effectiveness of psychopharmacological intervention may be because the “mental disorders” of eating disorders differ from those of patients with mental disorders. Thus, a rating scale that dissociates anxiety from other mental disorders in patients with mental disorders did not dissociate these disorders in 358 patients with anorexia nervosa [PS and others, manuscript submitted for Gutierrez and Birmingham (2018)].

The discovery that mental disorders are not distinct categories but vary along continuous dimensions was made long ago (Fisher, 1918; Porter, 2018), emphasized not long ago (Borsboom et al., 2011) and recently re-discovered (Brainstorm Consortium, Anttila et al., 2018; Plana-Ripoll et al., 2019; Schork et al., 2019). Hence, attempts to find genotype-phenotype correlations among eating disorders and mental disorders have yielded inconsistent results (Borsboom et al., 2011). Translating these results into treatments for eating disorders will be difficult (Breithaupt et al., 2018). This approach, which was launched 20 years ago in other contexts, has been marginally successful (Joyner and Paneth, 2019).

### The Standard Treatment

The standard treatment, cognitive behavioral therapy (CBT), assumes that eating disorders are maintained by cognitive processes. Even though CBT does not address the cause of eating disorders, it recognizes that the patients’ problems start with dieting (Slade et al., 2018). Launched for bulimia nervosa in 1981 (Fairburn, 1981), CBT is now recommended in the treatment guidelines for all eating disorders throughout the world [e.g., (NICE, 2017)].

Rather few patients have been treated with CBT in randomized controlled trials (RCT) (Slade et al., 2018). With a dropout rate ≈30%, which is generally expected and included in the power calculations of RCTs (Zipfel et al., 2014), a rate of remission <50% and a rate of relapse ≥30% within 1 year, ≤25% of the patients remain in remission at this point in time (Södersten et al., 2017).

Many more patients have been treated with CBT in general practice. For example, out of 683 patients referred to primary care for the treatment of bulimia within the United Kingdom healthcare system, 135 completed the treatment but although they improved, these patients did not remit (Knott et al., 2015). In Sweden, 15,411 patients were similarly treated in years 2012–2017 with a rate of remission of 18.4% at one year follow-up (Birgegård and Norring, 2019). There are no major differences between these outcomes and the outcomes in the specialized clinics in Sweden and other countries (Södersten et al., 2017, 2019).

What explains these low remission rates? Consider the most recent RCT in which 15 out of 36 patients (42%) went into remission from bulimia but not from anxiety (Poulsen et al., 2014). On the standard perspective, anxiety causes bulimia (Kaye et al., 2004) and it is unsurprising, therefore, that 5 of the 15 patients (33%) relapsed within 19 months. A new review found no “relevant new RCTs” and concluded that CBT is “an effective approach” (Slade et al., 2018), despite the fact that 22.2% of the patients dropped out, 33% relapsed and 39.3% received additional treatment during follow-up in the trial (Poulsen et al., 2014). Considering that there is no information of long-term outcomes, it should be possible to improve the effectiveness of CBT (Södersten et al., 2017; Slade et al., 2018). A new perspective might offer a start.

## The Evolutionary Perspective

A framework for anorexia nervosa, the prototypical eating disorder from which the other eating disorders emerge, was launched in 1996, with food restriction as the main cause (Bergh and Södersten, 1996). The neuroendocrine changes associated with this brain-based model have been reviewed (Bergh et al., 2002, 2013; Zandian et al., 2007; Södersten et al., 2008, 2016, 2017) and can be briefly updated as follows.

Because starvation has been the main threat in evolution it is fitting to paraphrase Dobzhansky: ”Nothing in the biology of anorexia makes sense except in the light of evolution” (Dobzhansky, 1973). And 36 years ago, it was realized that the conspicuous high physical activity of anorexia is a normal, evolutionary conserved response, i.e., foraging for food when food is in short supply (Epling et al., 1983). Later on, the evolutionary perspective was presented twice more (Guisinger, 2003; Södersten et al., 2008).

In fact, anorexia provides an example of the human homeostatic phenotype, as this concept emerged from the clinical observations and hypotheses of Bernard and the subsequent experimental verifications of Cannon (Södersten et al., 2008). This perspective has now been validated for brain function. Thus, the signaling molecules of the hypothalamic arcuate nucleus support the search for food, rather than eating (Ammar et al., 2000; Nergårdh et al., 2007; Chen et al., 2015; Dietrich et al., 2015; Burnett et al., 2016). The agouti-related protein neurons of this nucleus can monitor the availability of food in the environment, changing energy utilization from fat to carbohydrate (Chen et al., 2015; Burke et al., 2017; Cavalcanti-de-Albuquerque et al., 2019). Silencing these neurons eliminates the search for food but leaves chewing and swallowing unaffected (Thomas et al., 2018), replicating the effect of dopamine receptor blockade or depletion (Berridge et al., 1989; Bednar et al., 1992; Qian et al., 1998).

The search for food and eating behavior, chewing in particular, have dominated the evolution of the behavior and the anatomy of the individual, including the head and the brain (Lieberman, 2011, 2014; Ungar, 2017; Smith, 2018). “You are How you eat,” suggests the evolutionary biologist and even that we should “encourage [our children] to chew more gum” (Lieberman, 2011). And since it was first reported that chewing gum is relaxing 80 years ago (Hollingworth, 1939), it is now recognized that chewing gum promotes both physical and mental health (Fukushima-Nakayama et al., 2017). The neural engagement in these beneficial effects of chewing include the serotonin cells in the dorsal raphe nucleus in the brainstem and their projections to the prefrontal and orbitofrontal cortex (Ioakimidis et al., 2011). These serotonin neurons and the hypothalamic agouti-related protein neurons also activate dopamine neurons in the ventral tegmental area in the brainstem (Davis et al., 2011; Browne et al., 2019). Interestingly, activity in these mesolimbic dopamine neurons can functionally rearrange the connections within the prefrontal cortex (Kahnt and Tobler, 2017). Foraging for food has shaped these cortical and subcortical areas into an extended neural network, parts of which are differentially engaged dependent upon environmental conditions (Kolling et al., 2012; Pearson et al., 2014; Carlén, 2017; Korn and Bach, 2018). Dopamine, of course, plays roles in addition to the one(s) discussed here, some of which are important in evolution, including the management of threats (Miller et al., 2019).

It is well known that in evolution “men hunt and women gather” (Fessler, 2002; Gilby et al., 2017), but it is not yet known how these behavioral sex differences are related to the neural network of foraging. Research on the neuroscience of foraging often use economic rewards and choices, food rewards are less common (Kolling et al., 2012; Shenhav et al., 2016). However, it was observed long ago that the emergence of the prefrontal cortex in primate evolution coincided with improvement of the strategies for food foraging (overview in Genovesio et al., 2014). Gonadal hormone sensitive sex differences have since been demonstrated in the anatomy of the prefrontal cortex and these can be related to sexually dimorphic behavior (Clark and Goldman-Rakic, 1989; Evans and Hampson, 2015). On the evolutionary perspective, it is tempting, therefore, to speculate that these findings are related to the marked sex difference in the prevalence of anorexia nervosa.

The neurobehavioral responses to food deprivation and the corresponding genotype are evolutionarily conserved and consistent with the evolutionary perspective of anorexia nervosa (Södersten et al., 2008; Alvergne et al., 2010; Itskov et al., 2014; Gibson et al., 2015; Sato and Kawata, 2018).

### The Elusive Clinical Translation of the Neurobiology of Foraging

But rather than controlling behavior, the neural network just outlined is permissive; the cause of changes in eating behavior is outside of the individual (Södersten et al., 2011; Zandian et al., 2015). For example, the behavioral effects of experimental activation of the brainstem to prefrontal cortex part of the network in one environment are the opposite to the behavioral effect of the same experimental maneuver in another environment (Warden et al., 2012; Seo et al., 2019). Similarly, stimulating the brain with neuropeptide tyrosine makes a rat eat more food when food is continuously available but makes the rat forage for food and eat less food when the availability of food is restricted (Ammar et al., 2000; Nergårdh et al., 2007). These results support the proposed causal role of the environment in body weight regulation and suggest that neuropharmacological intervention may remain ineffective.

In normal circumstances, our biological propensity to eat as much as possible is counterbalanced by the need to forage for food (Södersten et al., 2011). But today the effort to find food is minimal and in the absence of internal controls people need external support in order not to lose control over body weight (Södersten et al., 2008).

### Eating Behavior in Treatment

In the clinic, we have therefore outsourced the control of eating behavior and body weight to a machine first described in 1996 (Bergh et al., 1996; Södersten and Bergh, 2014). The patients learn to eat assisted by visual feedback from a computer screen as described many times already and recently in an open access video (Esfandiari et al., 2018). But they are also treated with warmth, their physical activity is reduced and they are supported to resume their social activities (Bergh et al., 2002). An RCT demonstrated the treatments effectiveness (Bergh et al., 2002), which was confirmed by a description of the outcomes at 3 months intervals during treatment and 1, 2, 3, 6, 9, 12, 18, 24, 26, 48, and 60 months after remission in 1,428 patients treated in six clinics in four countries (Bergh et al., 2013). The rate of remission was estimated to 75% within on average 1 year of treatment and the rate of relapse was estimated to 10% (Bergh et al., 2013). Psychoactive drugs that had been prescribed prior to admission to treat mental symptoms were withdrawn while the patients remitted from these symptoms by re-learning how to eat (PS and others, manuscript submitted for Gutierrez and Birmingham, 2018).

## The Paradox of Standard Treatment

More patients go into remission in the long-term by re-learning how to eat than if treated with CBT (Södersten et al., 2017, 2019). The difference in outcome is unlikely due to difference in the state of the patients at admission. The published literature suggests the opposite; patients who are treated with standards of care are less serious ill at admission than patients whose eating behavior is treated (Södersten et al., 2016).

Considering the difference in outcomes, it is paradoxical that “the single most effective procedure in CBT” has long been recognized as “*the prescription of a pattern of regular eating”* (Fairburn et al., 1993). But because is unclear how this is achieved we have invited CBT-clinicians to use our method for treating eating behavior (Södersten et al., 2017).

## How to Eat

The biological, default pattern of eating behavior, a gradual decrease in the rate of eating over the course of a meal, was first described in experimental animals as: N = Kt^n^; where N = amount of food eaten at time t and K and n are constants (Skinner, 1930) and then modified as an exponential growth curve: f = c(l-e^-mt^); where f = amount of food eaten, c and m constants and t = time (Bousfield, 1933). A model of human eating behavior was presented as: y = kx^2^+lx; where y = amount of food eaten, k = change in the rate of eating over the course of the meal and l = initial rate of eating (Pudel, 1971). This model was subsequently confirmed (Kissileff et al., 1982). The recent suggestion that the model should predict outcomes and disclose mechanisms is based on 40 year old experimental results rather than the recent biology of foraging (Thomas et al., 2017). At present, the model remains descriptive, but as outlined here, it can be used in the treatment of eating behavior in patients with eating disorders.

With *k* < 0 in the model, Westerterp-Plantenga launched the term decelerated eating and with k≈0, she launched the term linear eating (Westerterp-Plantenga et al., 1990). If rats are deprived of food for 4 days, food intake decreases the linearity of eating increases (Bousfield and Elliott, 1934). Women respond in the same manner after merely skipping dinner (Levitsky and DeRosimo, 2010; Zandian et al., 2011).

Linear eaters eat less food yet feel increasingly full when eating at a reduced rate experimentally and they eat more food yet feel less full when eating at an increased rate experimentally (Zandian et al., 2009a). Thus, dieting, the main cause of anorexia, causes linear eating very rapidly and puts women at risk of losing control over food intake. These undesirable effects can be prevented by practicing eating at a decelerated rate (Zandian et al., 2009b). And when women transit from linear eating to decelerated eating their mental state normalizes (Zandian et al., 2009b), just as 737 patients remitted from their mental symptoms by re-learning how to eat (Bergh et al., 2013).

## The Eating Behavior of Anorexic Patients Treated to Remission

The derivative of the old model is a line but growth, including cumulative food intake, tapers off. We therefore re-launch the two-parameter asymptotic exponential curve as a minimally redundant model of eating behavior: y = a(1-e^-bt^), where y = amount of food eaten, a = hypothetical maximal food intake, b = change in the rate of eating and t = time (Bousfield, 1933).

Using non-linear regression (Bates and Chambers, 1992; R Nonlinear Regression, 2019), we describe the eating behavior of 17 women who were treated to remission from anorexia nervosa by practicing how to eat. Their mean (SD) age was 18.8 (3.7) years, they had been ill for 3.3 (2.2) years and had a Body Mass Index, BMI = 14.9 (1.0) at admission. The women went into remission in 359 (78) days, at a BMI = 19.8 (0.9). For a complete list of remission criteria, see (Bergh et al., 2002). Their eating behavior was compared to that of 17 healthy women, who were 23.6 (2.0) years old and had a BMI = 23.5 (1.5). The choice of 5 years older healthy women for comparison was based on the fact that patients who have been treated to remission are followed up for 5 years before they are considered cured (Bergh et al., 2002).

**FIGURE 1:**
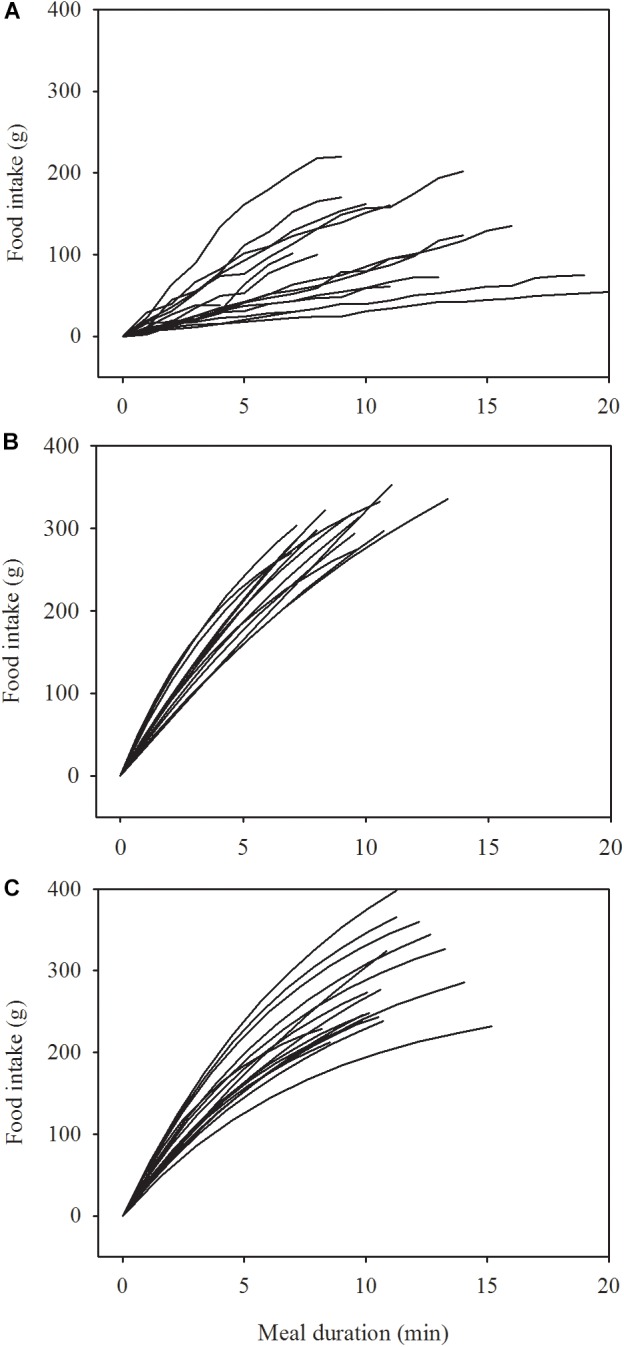
Change in the rate of eating in 17 women at admission **(A)** and at remission **(B)** after treatment of anorexia nervosa and in 17 healthy women **(C)**. Data in **A** are raw data collected at 1 min intervals. Data in **B,C** are modeled by a two-parameter asymptotic exponential curve, see text for details.

**Table 1** shows that the patients ate only little food, slowly, at admission, but when in remission, they ate somewhat more food than the healthy women and the duration of their meal was a little shorter. While the initial rate of eating among the anorexics in remission and the healthy women was similar, the rate of eating decreased over the course of the meal significantly more among the healthy women than among the women in remission. These differences in eating behavior emerge clearly in **Figure 1**. One of the patients continued eating for 37 min at admission, i.e., beyond the 20 min limit displayed (bottom graph in panel A). Three patients ate in a linear manner at remission and their curves are therefore omitted in panel B.

**Table 1:** Food intake, meal duration, initial rate of eating, change in rate of eating over the course of the meal (b) and hypothetical maximal food intake (a) in 17 women at admission for the treatment of Anorexia Nervosa (Adm AN) and at remission after treatment (Rem AN) and in 17 healthy women (Healthy).

Group	Food intake (g)	Meal duration (min)	Initial rate of eating (g/min)^a^	Change in rate of eating (b)	Hypothetical maximal food intake (a)
Adm AN	100 (72–160)^b^	10.4 (7.7–13.6)	8.4 (6.2–16.3)^b^	–	
Rem AN	307 (266–351)^c^	9.6 (8.0–10.6)^c^	44.3 (34.4–46.9)	0.06 (0–0.22)^d^	586 (377–684)
Healthy	268 (208–389)	10.7 (10.1–12.2)	39.1 (35.8–46.9)	0.12 (0.05–0.23)	374 (333–444)

Values are median (quartile range). See text for model of eating behavior. ^*a*^Derivative at time=1 min of the estimated function; ^*b*^*p* < 0.01 vs. Healthy or Rem AN; ^*c*^*p* = 0.06 vs. Healthy; ^*d*^*p* = 0.015 (Mann-Whitney *U*-test).

## Comments, Personal Insights and Opinions

While the anorexic women who practiced eating reached a BMI within the normal range and consumed a normal amount of food, their weight and their eating behavior was not the same as those of the healthy women. Our patients are followed for 5 years after treatment, including eleven appointments (Bergh et al., 2002, 2013) and, at present, we are examining if their physical characteristics and their eating behavior more closely resembles those of healthy women once they have completed the follow-up program. Yet, at the present state of knowledge, it is reasonable to suggest that patients with eating disorders should be offered the chance to practice eating using the device that has now restored the physical and mental health of hundreds of patients (Bergh et al., 2013; Södersten et al., 2017, 2019). Eating behavior thus treated makes it less important, albeit perhaps not unimportant, to treat cognitive processes (Södersten et al., 2017), although evidence that these interventions are redundant was presented 31 years ago (Freeman et al., 1988).

Practicing eating restores the levels of hormones thought to cause weight problems in obesity (Galhardo et al., 2012; Södersten et al., 2015), suggesting eating behavior control of hormonal secretion, i.e., the opposite causal relationship to the conventional homeostatic relationship (Lowell, 2019). The bidirectional relationship among brain and behavior, suggested by Darwin (1872) and confirmed in recent years (Woods, 1991; Ramsay and Woods, 2014), provides support for clinical translation of the present perspective.

In 1996 we suggested that eating disorders are eating disorders, rather than mental disorders, and that the patients therefore should practice eating (Bergh and Södersten, 1996; Bergh et al., 1996). At the time, it was thought that this was misplaced and even dangerous (Crisp, 1995), but today, 23 years later, no-one can treat patients with eating disorders in the Region of Stockholm unless a program for restoring their eating behavior is included in the treatment. Such overly long delays before evidence-based interventions are introduced into clinical practice are common (Kim et al., 2013). Policies to shorten the delay would be useful.

## Data Availability

The datasets generated for this study are available on request to the corresponding author.

## Ethics Statement

Data on eating behavior is part of treatment and the clinical files are kept in the register of the Mandometer Clinic, approved by the Regional Ethical Review Board of Stockholm. Patients entering treatment are informed verbally and in writing that their data might be used in research and if so, it will be anonymized. They are also informed that they can request that their data is not used and that they can leave the treatment any time without giving a reason. Written consent by the patients is not required for analysis of data collected in registries. The data from the healthy women were re-analyzed from a previous, ethically approved study (Zandian et al., 2009a).

## Author Contributions

PS launched the idea with all authors. UB undertook the statistical analysis and reviewed these with PS in detail and repeatedly. MZ was keeper of databases and clinical files and quality controller at the clinic. CB was a clinical director and supervised all treatments. All authors contributed intellectually to the content and to completing the final version of the manuscript which has been approved accordingly and wrote and reviewed the manuscript repeatedly.

## Conflict of Interest Statement

Complete openness concerning financial arrangements is intended here. UB and MZ declare that they have no financial interests related to this study. Our research is carried out at the Karolinska Institute, where PS is an emeritus professor. The research is translated clinically by Mando Group AB, a company started by PS and CB, who have 47.5% of the stock each. Professor Michael Leon of the University of California at Irvine has 5%. Mando Group AB contracts with the County Council of Stockholm every fifth year to treat patients with eating disorders. Mando Groups AB signed its first contract in 1997 with the County Council of Stockholm and, since then, its treatment is one of the standards of care treatments offered to the citizens of Stockholm. This arrangement is the same as when the County Council of Stockholm contracts with its own clinics to treat patients with all kinds of disease, including eating disorders. That is to say, the County Council of Stockholm provides eating-disorder services to the citizens of Stockholm both through a clinic of its own and through Mando Group AB. Until recently, there was a third provider of care for patients with eating disorders in Stockholm, which was a private clinic. Mando Group AB is the biggest provider of eating disorders services in Sweden as of 2019. All health care in Sweden is funded through the tax system; private pay is extremely uncommon. It should be added firstly, that Mando Group AB is in compliance with the recommendation of the International Committee of Medical Journal Editors on “Author Responsibilities-Conflicts of Interest” http://www.icmje.org/recommendations/browse/roles-and-responsibilities/author-responsibilities--conflicts-of-interest.html. Secondly, it should also be added that all profit that Mando Group AB has made has been re-invested in research and development and that there have been no dividends to stock owners. All of the above is declared in all manuscript submissions and thus far, journals have judged it necessary to publish only some of the details. It seems, however, that the potential ethical problem when scientists translate their research findings into the clinic in a company is not unlike that which arises when any scientist, in an academic setting is developing a theory and needs further economic funding for her/his work and may receive recognition and financial benefits for the work. The incentive is, in part, economic in this case as well and the ethical “problem” is similar in both cases. However, the more important incentive is the improvement of the treatment of patients with eating disorders. We are researchers working in an academic setting and like many other medical research institutes today, the Karolinska Institute encourages scientists to translate their research into the clinic in companies that aim to generate financial profits to be used for research and development (see https://issuu.com/karolinska_institutet/docs/ki_strategy2030_eng).
